# Prevalence of Pediatric Surgical Conditions Across Somaliland

**DOI:** 10.1001/jamanetworkopen.2018.6857

**Published:** 2019-01-11

**Authors:** Tessa Concepcion, Mubarak Mohamed, Shugri Dahir, Edna Adan Ismail, Dan Poenaru, Henry E. Rice, Emily R. Smith

**Affiliations:** 1 Duke Global Health Institute, Duke University, Durham, North Carolina; 2 Edna Adan University Hospital, Hargeisa, Somaliland; 3 Department of Pediatric Surgery, McGill University Health Centre, Montreal Children’s Hospital, Montreal, Quebec, Canada; 4 Department of Public Health, Robbins College of Health and Human Sciences, Baylor University, Waco, Texas

## Abstract

**Importance:**

Although surgical conditions are increasingly recognized as causing a significant health care burden among adults in low- and middle-income countries (LMICs), the burden of surgical conditions among children in LMICs remains poorly defined.

**Objective:**

To estimate the prevalence of pediatric surgical conditions across Somaliland using a nationwide community-based household survey.

**Design, Setting, and Participants:**

This cross-sectional study was conducted through a national community-based sampling survey from August through December 2017 in Somaliland. Participants were 1503 children surveyed using the Surgeons OverSeas Assessment of Surgical Need (SOSAS).

**Main Outcomes and Measures:**

The SOSAS survey contains 2 components, including a section on household demographics, deaths, and financial information and sections querying children’s history of surgical conditions.

**Results:**

In this cross-sectional study that included 1503 children (55.6% male; mean [SE] age, 6.4 [0.1] years), 221 surgical conditions were identified among 196 children, yielding a mean (SE) prevalence of pediatric surgical conditions of 12.2% (1.5%). Only 53 of these 221 surgical conditions (23.7%) had been surgically corrected at the time of the survey. The most common conditions encountered were congenital anomalies (33.8%) and wound-related injuries (24.6%). Nationally, an estimated 256 745 children have surgical conditions, with an estimated 88 345 to 199 639 children having unmet surgical needs.

**Conclusions and Relevance:**

Using national sampling, this study found that children have a high burden of surgical conditions in Somaliland. These data highlight the need for a scale-up of pediatric surgical infrastructure and resources to provide the needed surgical care for children in LMICs.

## Key Points

**Question:**

What is the prevalence of pediatric surgical conditions in Somaliland?

**Findings:**

In this cross-sectional study that included 1503 children in Somaliland, the prevalence of pediatric surgical conditions was 12.2%. Only 23.7% of surgical conditions had been corrected at the time of this study.

**Meaning:**

A scale-up of pediatric surgical infrastructure and resources to provide the needed surgical care for children in low- and middle-income countries is warranted.

## Teaser

This cross-sectional study measures the prevalence of pediatric surgical conditions across Somaliland using a nationwide community-based household survey.

## Introduction

Recent estimates indicate that 5 billion people, predominantly in low- and middle-income countries (LMICs), lack access to safe and affordable surgery,^1^ and surgical conditions contribute to up to 32% of the global disease burden.^2^ The World Health Organization, the World Bank, and the United Nations have all noted that access to adequate surgical care is essential to achieve the Sustainable Development Goals, which include health system strengthening and universal health coverage.^3,4,5^ Although addressing surgical needs has been shown to form an essential part of functioning health care systems, little priority has been given to addressing gaps in the surgical care for children.^6,7^

Children have surgical needs that are fundamentally different from those of adults.^8,9^ Congenital anomalies and injuries form a large portion of the overall surgical burden and disproportionately affect children.^10,11,12,13,14^ Pediatric surgical care requires specific infrastructure, workforce, and resources that differ from adult care.^15,16,17^ Many areas of surgical care for children are cost-effective and in appropriate settings can provide financial protection against medical impoverishment to families in need.^18,19^

Existing data suggest a large burden of pediatric surgical conditions in LMICs, with reports ranging from 10% to 85% of children in sub-Saharan Africa having a surgical condition.^14,20,21^ However, precise estimates on the burden of surgical conditions among children remain limited due to lack of high-quality data, reliance on small cohort studies, use of institutional-based surveys (which do not capture out-of-hospital disease), and a focus on urban areas.^20,21^ Surgical conditions in children have been largely left out of contemporary national health assessments, limiting the ability to develop inclusive, effective health care policies.^7,9^

Although several recent studies have estimated the prevalence of surgical conditions in LMICs, most existing studies do not focus on pediatric conditions, and few assess surgical conditions across an entire country.^22,23,24,25,26,27^ Our objective was to estimate the national burden of surgical disease among children in Somaliland using a nationwide community-based household survey. The long-term goal of the study is to provide a foundation for scale-up modeling and capacity building to support pediatric surgical care in Somaliland.

## Methods

Institutional review board approval was granted from Duke University. Because Somaliland does not have a national institutional review board, a letter of approval was granted from the Somaliland Ministry of Health. Participants in the community survey gave verbal informed consent for study participation. A parent or guardian provided consent for all children younger than 16 years, and children aged 12 to 15 years provided assent. For most children enrolled, parents answered all questions in the survey. This study followed the Strengthening the Reporting of Observational Studies in Epidemiology (STROBE) reporting guideline.

### Setting

This study took place in Somaliland, a country in the Horn of Africa that (although not recognized as an independent state) has achieved relative stability after separation from Somalia. Since 1991, Somaliland has set up an autonomous government, with several presidential elections.^28^ The country has a gross domestic product per capita of $348, classifying it as a low-income country by World Bank income groups and the fourth poorest in the world.^29^ Mortality rates of infants and those younger than 5 years are 109 and 180 per 100 000, respectively.^30^ These rates are more than twice as high as overall mortality rates in sub-Saharan Africa (55 and 83 per 100 000, respectively).^31,32^ Only 17% of Somalilanders live within 2 hours of a surgeon.^33^ Somaliland includes the following 6 regions: Awdal, Maroodi Jeex, Sahil, Sanaag, Sool, and Togdheer. Of the total population of 4 million people, approximately 50% are children younger than 16 years.^34^

### Participants

From August through December 2017, we collected data on the burden of surgical conditions in children using community-based national sampling across Somaliland. We used the Surgeons OverSeas Assessment of Surgical Need (SOSAS) survey, a validated, cluster-based, cross-sectional survey designed to determine the burden of surgical conditions within a community.^22,23,25,35^ The survey can be found online (https://www.surgeonsoverseas.org/resources). All survey methods comply with the American Association for Public Opinion Research (AAPOR) reporting guideline for survey studies.^36^ We used the SOSAS survey in 871 households, with 2 children assessed per household via paper data collection. Two children were randomly selected from the household by assigning each child a number and using a smartphone random number generator application. This sample size was calculated using a pediatric surgical disease prevalence of 19%, the estimated prevalence of pediatric surgical disease in other LMICs from prior studies.^22,24,25,34^ We used estimates for response rate, eligibility rate, and design effect similar to previous SOSAS survey investigations.^35^ Although the SOSAS survey was originally designed to include both children and adults, our study focused on surgical conditions in children up to age 15 years.

Survey clusters were randomly selected in a 2-stage process with a probability adjustment for population size by region. Data from 2005 and 2014 Somalia censuses were used to estimate populations of Somaliland’s 6 regions.^37,38^ The capital city of Hargeisa comprises 40% of the total population of Somaliland and was considered a separate region for the sampling strategy. Sampling strata were population weighted at the regional level to include representation of all 6 regions. Cities and semicities were given a weight of 2, and villages were given a weight of 1 in the selection. Within each household, up to 2 children were enrolled in the study.

### Data Collection

The SOSAS survey was translated into Somali and administered by a pair of enumerators per household. Each enumerator pair included a public health professional with survey collection experience and a nurse, both from Somaliland and fluent in Somali and English. The SOSAS survey contains the following 2 components: (1) a section on household demographics, deaths, and financial information and (2) sections querying children’s history of surgical conditions. Health facilities were defined according to the SOSAS survey guidelines^39^ as primary (a facility without a functioning operating room), secondary (a facility with a functioning operating room), and tertiary (a facility with a functioning operating room and a surgical specialist, such as a general surgeon, orthopedic surgeon, or pediatric surgeon). Because many families did not know the difference between secondary and tertiary hospitals, these 2 categories were combined in our analysis.

In the section querying a child’s history of surgical conditions, the responder was first asked if the child has ever had “a wound, burn, mass/goiter, deformity, or problem with [specific problems associated with that body region].” If so, follow-up questions were asked about condition specifics, treatment sought, and disability. We defined surgical conditions using *The Lancet* Commission on Global Surgery as “any disease, illness, or injury in which surgical care can potentially improve the outcome.”^1^^(p6)^ A surgical need was self-reported by the parents or guardians of the children as a condition that required surgical consultation. Before data analysis, conditions were confirmed as surgical by a pediatric surgeon (H.E.R.) not involved with the data collection. The lifetime prevalence of surgical conditions was determined as the rate of children who reported a surgical condition at some point in their life. Respondents were asked if any type of care was provided for these surgical conditions, including care provided at a health care facility (defined as care provided by a physician or nurse at a health facility) or traditional care (defined as care provided by a traditional healer outside of a health care facility). A major procedure was defined as one that requires regional or general anesthesia; minor procedures were defined as dressings, wound care, punctures, suturing, and incision and drainage.^39^

### Statistical Analysis

For data analysis, we weighted households and individuals based on regional populations using census data^37^ and pediatric proportion estimates.^34^ Data were analyzed using SAS (version 9.4; SAS Institute Inc) and Microsoft Excel 2010 (Microsoft Corp). All data were analyzed incorporating proportional-to-size methods, cluster-based sampling, and design weights based on sampling fractions. Household and child demographic data were analyzed through survey frequencies (with weighted percentages), medians (with associated interquartile ranges [IQRs]), or mean (SE). Demographics were compared across regions using the Wald χ^2^ statistic for categorical variables and regression statistics for continuous variables. Missing values were included in analysis of frequency and weighted percentages but were excluded from analysis of *P* values due to low numbers. Household-, child-, and condition-specific data were compared between children whose caregivers reported a surgical condition and those who did not, as well as between children who did not seek health care, sought health care but did not receive a surgical procedure, or received a surgical procedure. Significance testing was set at 2-sided *P* < .05.

## Results

In this study, there were 1503 children surveyed for the prevalence of pediatric surgical conditions. Of these children, 43.5% (n = 667) were female, and 55.6% (n = 836) were male. The mean (SE) age was 6.4 (0.1) years, with 5.0% (n = 77) younger than 1 year, 43.0% (n = 650) aged 1 to 5 years, 33.0% (n = 490) aged 6 to 10 years, and 19.0% (n = 286) aged 11 to 15 years. We found a total of 221 surgical conditions among 196 children, yielding a mean (SE) prevalence of pediatric surgical conditions of 12.2% (1.5%). For children with surgical conditions, the mean (SE) age was 6.8 (0.4) years, with 6.6% (n = 15) being younger than 1 year, 36.3% (n = 78) being aged 1 to 5 years, 36.4% (n = 81) being aged 6 to 10 years, and 20.7% (n = 47) being aged 11 to 15 years.

### Demographics of Households

In this study, 871 families were asked to participate, and 33 declined participation, resulting in 838 families. A total of 1503 children were included for analysis because not all families had 2 eligible children (ie, children were older than 15 years). Three hundred ninety-nine of these families were from rural areas (Table 1). The median household size was 5.8 members (IQR, 4.0-7.9 members), and the median number of children younger than 16 years was 2.6 (IQR, 1.2-4.2). In total, 53.7% (n = 2997) of household members were younger than 16 years; of these children, those aged 1 to 5 years (18.9% [n = 1090]) and 6 to 10 years (18.2% [n = 1002]) represented the highest proportions of age groups.

**Table 1.** Household Demographics Among 838 Families^a^

Variable	Value
**Demographic Information**	
Village type, No. (%)	
Rural	399 (51.0)
Urban	439 (49.0)
Household size, median (IQR)	5.8 (4.0-7.9)
No. of children per household, median (IQR)	2.6 (1.2-4.2)
Household age, y, No. (%)	
<1	146 (2.4)
1-5	1090 (18.9)
6-10	1002 (18.2)
11-15	759 (14.1)
>15	2623 (46.3)
**Health Facility Information**	
Primary	
Type, No. (%)	
Private	270 (31.1)
Public	452 (56.1)
Unknown/missing	116 (12.8)
Type of transport, No. (%)^b^	
Public transport	661 (82.0)
Car	86 (9.0)
On foot	22 (2.4)
Carried	2 (0.0)
Unknown/missing	68 (6.6)
Cost of transport, median (IQR), $^c^	0.90 (0.40-2.60)
Secondary and Tertiary (n = 1678)	
Type, No. (%)	
Private	394 (33.3)
Public	657 (66.7)
Unknown/missing	646 (NA)
Travel time to facility	
Median (IQR), h	0.9 (0.5-1.9)
≤2 h, No. (%)	897 (76.9)
>2 h, No. (%)	208 (23.1)
Unknown/missing, No. (%)	592 (NA)
Type of transport, No. (%)^b^	
Public transport	642 (54.2)
Car	317 (31.9)
On foot	139 (13.2)
Carried	5 (0.7)
Unknown/missing	594 (NA)
Cost of transport, median (IQR), $^c^	9.20 (2.90-125.60)

Abbreviations: IQR, interquartile range; NA, not applicable.

^a^ Percentages were weighted as described in the text. Thirty-three families declined participation for the following reasons: no time (65.6%), no perceived benefit (21.9%), not willing (9.4%), and other (sick child) (3.1%).

^b^ Other options (motorcycle, bicycle, boat, or animal) had no responses.

^c^ If cost exceeded $0.00. For families who reported cost in Somaliland shillings, the current exchange rate of $1 to 10 000 Somaliland shillings was used.

Travel time, cost, and type of transport varied between primary and secondary or tertiary health facilities.^39^ Families reported that the closest secondary or tertiary health provider was at a public health facility (66.7% [n = 657]) within a median travel time of 0.9 hours (IQR, 0.5-1.9 hours). Most families (76.9% [n = 897]) reported being within 2 hours of a secondary or tertiary health facility. Families reported traveling to secondary or tertiary health facilities mostly by public transport (54.2% [n = 642]), with the median cost of transport being $9.20 (IQR, $2.90-$125.60).

### Demographics of Children

For children 6 years and older who had data on education, 34.7% (n = 293) had no education, 61.5% (n = 451) had primary school education, and 3.0% (n = 25) had secondary school education. Most children (76.9% [n = 897]) reported that they lived within 2 hours of a secondary or tertiary health facility, while the mean (SE) cost of transportation to this facility was $92.40 ($5.86) (Table 2).

**Table 2.** Demographic Characteristics of Children Stratified by Surgical Condition, Health Care Seeking, and Surgical Treatment Status Among 1503 Children^a^

Variable	Value			*P* Value	Surgical Condition,^b^ Value			*P* Value
	Total	No Surgical Condition	Surgical Condition^b^		No Health Care, No Surgery	Health Care, No Surgery	Received Surgery	
No. (%)	1503 (100)	1307 (87.8)	196 (12.2)	NA	64 (33.6)	95 (42.7)	53 (23.7)	NA
Age, mean (SE), y	6.4 (0.1)	6.4 (0.1)	6.8 (0.4)	.22	6.5 (0.7)	7.1 (0.5)	6.6 (0.2)	.44
<1	77 (5.0)	65 (4.8)	15 (6.6)	.43	7 (27.2)	6 (5.5)	2 (1.5)	.52
1-5	650 (43.0)	580 (43.8)	78 (36.3)		25 (36.4)	28 (32.2)	21 (41.5)	
6-10	490 (33.0)	417 (32.5)	81 (36.4)		18 (29.8)	38 (39.1)	23 (44.7)	
10-15	286 (19.0)	245 (18.9)	47 (20.7)		14 (21.7)	23 (23.2)	7 (12.3)	
Sex, No. (%)								
Male	836 (55.6)	719 (55.8)	128 (60.0)	.33	34 (56.1)	54 (55.6)	33 (68.8)	.21
Female	667 (43.5)	588 (44.2)	92 (40.0)		29 (43.9)	41 (44.4)	20 (31.2)	
No. of children per family, mean (SE)	3.7 (0.1)	3.7 (0.1)	3.9 (0.1)	.09	3.5 (0.2)	4.1 (0.2)	4.3 (0.1)	.006
Village type, No. (%)								
Rural	721 (51.4)	644 (53.0)	85 (39.0)	.12	36 (56.5)	35 (36.9)	11 (21.1)	.26
Urban	782 (48.6)	663 (47.0)	136 (39.0)		28 (43.5)	60 (63.1)	42 (78.9)	
Region, No. (%)								
Maroodi Jeex	874 (40.5)	746 (39.2)	147 (52.3)	.11	32 (32.3)	62 (53.5)	45 (73.8)	NA
Togdheer	217 (18.2)	199 (18.8)	18 (12.2)		4 (7.8)	9 (14.1)	5 (16.4)	
Awdal	167 (17.0)	149 (17.5)	23 (14.4)		15 (30.1)	8 (11.1)	NA	
Sanaag	109 (13.2)	101 (14.0)	8 (7.0)		6 (16)	2 (10.7)	NA	
Sool	69 (8.5)	58 (8.1)	11 (10.1)		4 (11.1)	5 (10.7)	2 (8.6)	
Sahil	67 (2.6)	54 (2.4)	14 (4.1)		3 (2.7)	9 (6.3)	1 (1.2)	
Secondary and tertiary facility travel time, No. (%)								
≤2 h	897 (76.9)	776 (77.6)	121 (72.8)	.43	23 (47.5)	56 (84.8)	35 (81.3)	.03
>2 h	208 (23.1)	177 (22.4)	31 (27.2)		17 (52.5)	10 (15.2)	4 (18.7)	
Cost of transport, mean (SE), $^c^								
Primary care	2.70 (0.84)	2.70 (0.86)	2.80 (0.86)	.88	1.90 (1.50)	4.00 (1.10)	2.20 (0.20)	<.001
Secondary/tertiary care	92.40 (5.86)	45.90 (21.15)	198.10 (27.80)	.004	3.00 (0.00)	28.40 (2.80)	239.00 (11.50)	<.001

Abbreviation: NA, not applicable.

^a^ Percentages were weighted as described in the text. For 9 surgical conditions, the children did not report if they received health care or surgery.

^b^ Of the 196 children, 173 had 1 surgical condition, 42 had 2 surgical conditions, and 6 had 3 surgical conditions, resulting in 221 reported surgical conditions. Variables may sum to more than 100% due to multiple conditions reported.

^c^ If cost exceeded $0.00.

### Demographics by Surgical Condition, Health Care**–**Seeking Behavior, and Surgical Treatment

We found a total of 221 surgical conditions among 196 children, yielding a mean (SE) prevalence of pediatric surgical conditions of 12.2% (1.5%). Compared with children who did not have surgical conditions, children with surgical conditions were less often considered generally healthy and had significantly more health facility visits. Of the 221 surgical conditions, 64 children (33.6%) reported not seeking health care, 95 children (42.7%) reported seeking health care but not receiving surgery, and only 53 children (23.7%) reported having received major or minor surgery for their condition at the time of the survey. The most common conditions encountered were congenital anomalies (33.8%) and wound-related injuries (24.6%). There was a significant difference in the number of children per family for health care status, with those receiving surgery having the most children per family (4.3 children). More than one-half (52.5% [n = 17]) of those who did not seek health care or receive surgery lived more than 2 hours away from secondary or tertiary facilities, whereas those who sought health care or received surgery mostly lived within 2 hours (84.8% [n = 56] and 81.3% [n = 35], respectively) (*P* = .03).

### Condition Specifics by Health Care**–**Seeking Behavior and Surgical Treatment

We found several trends in the type of surgical conditions, health care–seeking behaviors, and surgical treatment in children with surgical conditions (Table 3). The most common conditions were congenital deformities (34.7% [n = 70]), followed by wound-related injuries (25.3% [n = 51]), other wounds (11.9% [n = 25]), burns (11.3% [n = 30]), acquired deformities (10.5% [n = 25]), masses (3.2% [n = 8]), and gastrointestinal problems (2.9% [n = 7]). More children who received surgery also sought traditional health care (33.5% [n = 14]) than children who did not seek any health care (21.4% [n = 13]) or did not receive surgery (13.7% [n = 13]). Children who received surgery typically had injury-related wounds (38.5% [n = 20]) due to falls (62.3% [n = 17]). Children with masses had the highest proportion receiving surgery (57.0%), while no children with acquired deformities received surgery, and less than one-fifth of children with congenital deformities or gastrointestinal problems received surgery (19.7% and 14.3%, respectively) (Figure).

**Table 3.** Surgical Condition Specifics by Health Care Seeking and Surgical Treatment Status Among 221 Surgical Conditions^a^

Variable	No. (%)				*P* Value
	Total	No Health Care, No Surgery	Health Care, No Surgery	Received Surgery	
No. of children interviewed	1503	NA	NA	NA	NA
No. of children with surgical conditions	196 (12.2)	NA	NA	NA	NA
No. of surgical conditions	221	64 (33.6)	95 (42.7)	53 (23.7)	NA
Surgical condition present now (yes)	128 (62.2)	42 (72.4)	69 (74.8)	14 (26.5)	.17
Timing of onset					
<1 mo	17 (7.2)	4 (7.0)	8 (7.9)	4 (6.1)	.39
1-12 mo	53 (22.7)	16 (22.6)	20 (20.3)	15 (25.5)	
1-3 y	48 (22.1)	15 (21.8)	15 (15.1)	17 (35.7)	
3-7 y	58 (29.5)	17 (30.1)	31 (34.4)	9 (19.6)	
>7 y	41 (18.6)	12 (18.4)	21 (22.3)	8 (13.1)	
Surgical condition					
Deformity, congenital	70 (34.7)	33 (52.1)	21 (24.7)	14 (29.1)	NA
Wound, injury related	51 (25.3)	9 (16.4)	21 (25.4)	20 (38.5)	
Burn	30 (11.3)	5 (6.2)	18 (14.7)	5 (10.2)	
Wound, not injury related	25 (11.9)	6 (10.8)	11 (12.2)	7 (12.5)	
Deformity, acquired	25 (10.5)	9 (10.6)	15 (16.8)	NA	
Masses	8 (3.2)	2 (3.9)	1 (0.2)	5 (7.9)	
GI conditions	7 (2.9)	NA	6 (6.1)	1 (1.8)	
Type of injury					
Fall	36 (48.1)	7 (43.6)	11 (38.7)	17 (62.3)	.17
Fire or explosion	24 (33.4)	6 (38.4)	10 (37.1)	8 (27.4)	
Other^b^	7 (10.6)	1 (9.3)	5 (18.4)	1 (3.4)	
Vehicle crash	7 (7.9)	2 (8.7)	2 (5.9)	2 (6.8)	
Traditional health care					
No	176 (79.3)	50 (78.6)	82 (86.3)	39 (66.5)	.06
Yes	40 (20.7)	13 (21.4)	13 (13.7)	14 (33.5)	
Disability associated with surgical condition					
The condition is not disabling	130 (64.0)	32 (50.4)	58 (66.2)	37 (80.6)	.58
I feel ashamed	7 (4.1)	1 (2.8)	5 (6.4)	1 (2.1)	
I’m not able to work like I used to	7 (3.3)	2 (4.3)	1 (1.1)	4 (6.0)	
I need help with transportation and daily living	49 (28.6)	23 (42.5)	21 (26.3)	4 (11.3)	

Abbreviations: GI, gastrointestinal; NA, not applicable.

^a^ Percentages were weighted as described in the text. For 9 surgical conditions, the children did not report if they received health care or surgery.

^b^ Other injury options included gunshot, stab or cut, and bite or animal attack.

**Figure.**
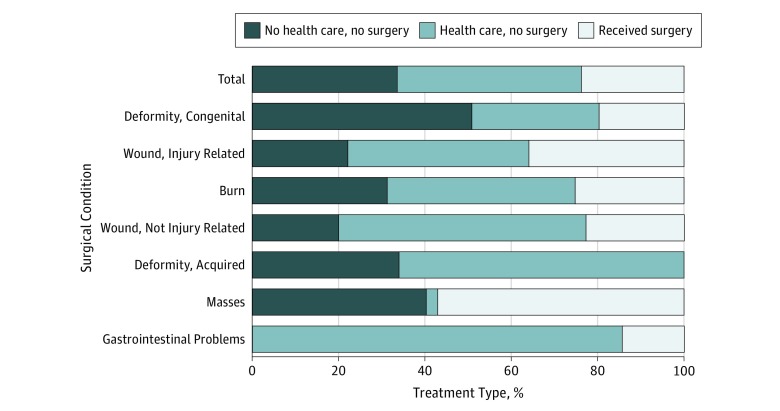
Ratios of Type of Health Care Received by Somaliland Children Stratified by Condition Type Among 196 children, 221 surgical conditions were identified.

### Surgical Conditions Stratified by Region

There were several trends among surgical conditions (n = 221) when stratified by region (Table 4). The region with the highest proportion of surgical conditions was Sahil (19.4% [n = 13]), followed by Sool (16.1% [n = 11]), Maroodi Jeex (15.1% [n = 128]), Awdal (9.6% [n = 18]), Togdheer (9.1% [n = 18]), and Sanaag (7.2% [n = 8]) (*P* = .03). More children reporting surgical conditions were 5 years or younger in Awdal (53.3% [n = 11]), Sahil (56.8% [n = 8]), and Sanaag (63.0% [n = 5]), while the children were older than 5 years in Maroodi Jeex (61.1% [n = 89]), Sool (73.8% [n = 8]), and Togdheer (55.0% [n = 10]) (*P* = .008). Congenital deformities were the most common condition type in all regions except Maroodi Jeex and Togdheer, where wound-related injuries composed 27.8% (n = 38) and 35.4% (n = 6), respectively. Most children did not seek health care in Awdal (68.2% [n = 15]) and Sanaag (74.6% [n = 6]); in these same regions, no child received a surgical procedure for his or her condition.

**Table 4.** Surgical Condition Specifics Stratified by Region Among 221 Surgical Conditions in 1503 Children Interviewed^a^

Variable	No. (%)						*P* Value
	Awdal	Maroodi Jeex	Sahil	Sanaag	Sool	Togdheer	
No. of children with surgical conditions (n = 196)	18 (9.6)	128 (15.1)	13 (19.4)	8 (7.2)	11 (16.1)	18 (9.1)	.03
Age, y							
≤5	11 (53.3)	58 (38.9)	8 (56.8)	5 (63.0)	3 (26.2)	8 (45.0)	.008
>5	12 (46.7)	89 (61.1)	6 (43.2)	3 (37.0)	8 (73.8)	10 (55.0)	
Generally healthy							
No	9 (31.1)	42 (30.1)	3 (21.6)	1 (13.0)	1 (9.9)	1 (6.7)	.27
Yes	14 (68.9)	105 (69.9)	11 (78.4)	6 (74.7)	10 (90.1)	17 (93.3)	
Surgical condition present now (yes)	23 (100.0)	67 (42.4)	11 (78.6)	7 (87.7)	8 (70.4)	12 (67.5)	NA
Surgical condition							
Deformity, congenital	16 (66.3)	33 (19.9)	7 (49.8)	4 (49.3)	5 (45.9)	5 (31.0)	NA
Wound, injury related	1 (3.4)	38 (27.8)	NA	3 (38.4)	3 (26.2)	6 (35.4)	
Burn	4 (19.4)	15 (10.1)	1 (7.3)	NA	1 (9.9)	4 (18.5)	
Wound, not injury related	1 (3.4)	24 (16.3)	4 (28.6)	NA	NA	1 (6.7)	
Deformity, acquired	NA	19 (13.1)	2 (14.3)	NA	2 (18.0)	2 (8.4)	
Masses	NA	7 (4.4)	NA	1 (12.3)	NA	NA	
GI conditions	NA	7 (5.4)	NA	NA	NA	NA	
Health care sought							
No	15 (68.2)	32 (20.1)	3 (21.6)	6 (74.6)	4 (36.1)	4 (20.8)	.34
Yes	8 (31.8)	111 (76.8)	11 (78.4)	2 (25.4)	7 (63.9)	14 (79.2)	
Traditional health care							
No	19 (80.8)	116 (77.5)	13 (92.7)	7 (87.0)	7 (87.0)	7 (87.0)	.58
Yes	4 (19.2)	26 (18.6)	1 (7.3)	1 (13.0)	1 (13.0)	1 (13.0)	
Type of care received							
No health care, no surgery	15 (68.2)	32 (21.2)	3 (23.2)	6 (74.6)	4 (36.1)	4 (20.8)	NA
Health care, no surgery	8 (31.8)	62 (44.6)	9 (69.2)	2 (25.4)	5 (44.2)	9 (48.2)	
Received surgery	NA	45 (34.2)	1 (7.6)	NA	2 (19.7)	5 (31.0)	

Abbreviations: GI, gastrointestinal; NA, not applicable.

^a^ Percentages were weighted as described in the text. For 9 surgical conditions, the children did not report if they received health care or surgery.

## Discussion

Surgical care is increasingly recognized as an essential component of a functional health system. With one of the highest infant mortality rates in the world and recent civil conflict in Somaliland, it is essential to accurately identify the burden of surgical conditions in the population, particularly among the vulnerable population of children.^1,40,41,42,43^ Before the present study, there were no published reports to our knowledge regarding the burden of surgical disease in children of Somaliland. Using a national community-based sampling survey, we found that 12.2% of children in Somaliland have a surgical condition. By extrapolating to the national population, an estimated 256 745 children across the country have surgical conditions, and 76.3% of these conditions are untreated. An estimated 88 345 to 199 639 children have unmet surgical needs.

The number of children with surgical conditions who remain untreated (ie, unmet surgical need) appears to be large, although it is difficult to estimate precisely. Unmet need refers to the rate of children with a surgical condition who did not obtain necessary care. The rate of children receiving necessary care was difficult to measure using the SOSAS survey because care may involve surgical consultation only, nonoperative surgical care, or a surgical procedure. Although children with surgical conditions do not always require a surgical procedure,^20^ the presence of surgical conditions generally requires the expertise of a surgically trained provider.^44^ Because we do not know if the type of health care involved a surgeon, we chose to report the unmet need as a range from children who did not seek any health care (definitely unmet need) to children who did not receive a surgical procedure (possibly unmet need). However, given the limited health system infrastructure for surgical care in Somaliland, the true unmet need likely lies at the higher end of the range.

Moving forward, we suggest measuring the receipt of surgical care according to the Three Delays Model as detailed by *The Lancet* Commission on Global Surgery, including delays in seeking care, reaching care, and receiving care.^1^ The unmet need could be stratified according to the care continuum and aid in planning targeted intervention programs. Using this model in the present study, we estimate that 42.7% of children with surgical conditions sought some form of health care but did not receive a surgical procedure. Families listed lack of money, limited transportation, and absence of perceived need among reasons for not receiving surgery, which align with several previous studies in LMICs.^1,45,46,47^ Although information on the quality and type of surgical care sought is not collected in a granular fashion using the SOSAS survey, the type of surgical care sought by families represents an important avenue for investigation in task shifting and health system planning.

We found several differences in surgical care across the regions of Somaliland. Almost one-fifth of children in the regions of Sahil and Sool had a surgical condition, and no children in the regions of Awdal and Sanaag received a surgical procedure. These rural regions are far from secondary or tertiary hospitals, and transportation to urban areas can take 24 hours or more and cost up to several hundred dollars. In our study, children in rural regions also had higher rates of congenital deformities, whereas children in urban areas had higher rates of injuries. In addition, urban cities in Somaliland are crowded, and dwellings are small, increasing the risk for injury from burns and explosions in the home.^48,49,50,51,52^ Despite these regional differences, there was a uniform unmet need for surgical care across Somaliland. Distance and cost are common barriers to health care across LMICs, particularly for surgical disease.^1,45,46,53^ *The Lancet* Commission on Global Surgery^1^ has proposed a target of at least 80% coverage of essential surgical and anesthesia services per country by 2030. Because poverty and unemployment are higher among rural areas in LMICs,^29^ addressing these underlying determinants is essential to improve surgical access for children.

We found that the prevalence of pediatric surgical conditions in Somaliland is similar to the prevalences reported in Rwanda (11.8%), Sierra Leone (27.5%), Uganda (17.1%), and Nepal (17.6%).^21,22,23,24,26,34^ However, the rate of unmet need in Somaliland is higher than other reported rates (70.3% in Sierra Leone, 64.9% in Uganda, 54.3% in Rwanda, and 41.8% in Nepal^34^). The types of surgical conditions in Somaliland also differ from those in these countries. In previous SOSAS survey investigations, burns (47%), deformities (21%), and masses (20%) were the most common surgical conditions in children.^34^ In the present study, congenital deformities and injury-related wounds were the most common conditions. A weakness of the SOSAS survey is its inability to identify “unseen” surgical conditions, such as cancers and masses.^35,39^ The high number of congenital deformities reported in Somaliland could result from a number of factors, although our study did not specifically assess the etiology of surgical conditions. Previous studies^54,55,56,57,58,59^ have identified lack of folic acid, high maternal age, and limited antenatal clinic visits as significant risk factors for congenital anomalies.

### Limitations

There are several limitations to our study, some of which are common to community-based health surveys.^22,23,24,26,34,39,60^ The enumerators used to assist with the data collection were medical professionals but not surgeons, raising concerns that they may not have appropriately recognized surgical disease. However, a pediatric surgeon reviewed all results and confirmed the suspected surgical conditions. A well-described limitation of the SOSAS survey is the use of self-reporting of surgical conditions. However, in a validation study^23^ in Nepal, the SOSAS survey was compared with a visual examination and demonstrated high concordance with participant self-reporting. There is also the risk of recall bias in the present study because parents with many children may not remember surgical conditions for all of their children, especially the older ones. Contextually, Somaliland has a large nomadic population that is unlikely to have received equitable representation in this survey. Nomadic families often live in an *aqal*, a dome-shaped, collapsible hut made from poles covered by hides or woven fiber mats. These types of households were included within the village portion of the survey but may have been underrepresented in urban areas because they are often found in the outskirts of towns and thus likely were not selected for inclusion. The SOSAS survey is limited in its ability to provide policy guidance for health system planning. Although the survey is limited in granular detail of health care provision (eg, outcomes of health care visits), it provides an overall assessment of pediatric surgical conditions. Surgical condition prevalence is a critical factor (but only a singular factor) in policy development. Analysis of other health system elements, such as workforce, infrastructure, finance, and economics, is also essential to develop rational policy to improve surgical care of children.

## Conclusions

Using a national community-based sampling study, we found that children in Somaliland have a high burden of surgical conditions, with most of these needs being unmet and inequitably concentrated in rural areas of the country. This tremendous burden of disease and high rate of unmet surgical care highlight the need for a scale-up of pediatric-specific infrastructure, resources, and workforce to provide the needed surgical care. Congenital deformities and injury-related conditions comprised a large portion of the surgical need, which provide further opportunities for screening programs and prevention strategies to improve children’s health.
